# D-Serine: Potential Therapeutic Agent and/or Biomarker in Schizophrenia and Depression?

**DOI:** 10.3389/fpsyt.2019.00025

**Published:** 2019-02-06

**Authors:** Mary-Anne B. MacKay, Maryana Kravtsenyuk, Rejish Thomas, Nicholas D. Mitchell, Serdar M. Dursun, Glen B. Baker

**Affiliations:** ^1^Neurochemical Research Unit and Bebensee Schizophrenia Research Unit, Department of Psychiatry, University of Alberta, Edmonton, AB, Canada; ^2^Neuroscience and Mental Health Institute, University of Alberta, Edmonton, AB, Canada

**Keywords:** D-serine, D-amino acids, schizophrenia, depression, serine racemase, D-amino acid oxidase

## Abstract

D-Serine is a potent co-agonist at the NMDA glutamate receptor and has been the object of many preclinical studies to ascertain the nature of its metabolism, its regional and cellular distribution in the brain, its physiological functions and its possible clinical relevance. The enzymes involved in its formation and catabolism are serine racemase (SR) and D-amino acid oxidase (DAAO), respectively, and manipulations of the activity of those enzymes have been useful in developing animal models of schizophrenia and in providing clues to the development of potential new antipsychotic strategies. Clinical studies have been conducted in schizophrenia patients to evaluate body fluid levels of D-serine and/or to use D-serine alone or in combination with antipsychotics to determine its effectiveness as a therapeutic agent. D-serine has also been used in combination with DAAO inhibitors in preclinical investigations, and interesting results have been obtained. Genetic studies and postmortem brain studies have also been conducted on D-serine and the enzymes involved in its metabolism. It is also of considerable interest that in recent years clinical and preclinical investigations have suggested that D-serine may also have antidepressant properties. Clinical studies have also shown that D-serine may be a biomarker for antidepressant response to ketamine. Relevant to both schizophrenia and depression, preclinical and clinical studies with D-serine indicate that it may be effective in reducing cognitive dysfunction.

## Introduction

**Figure 1:**
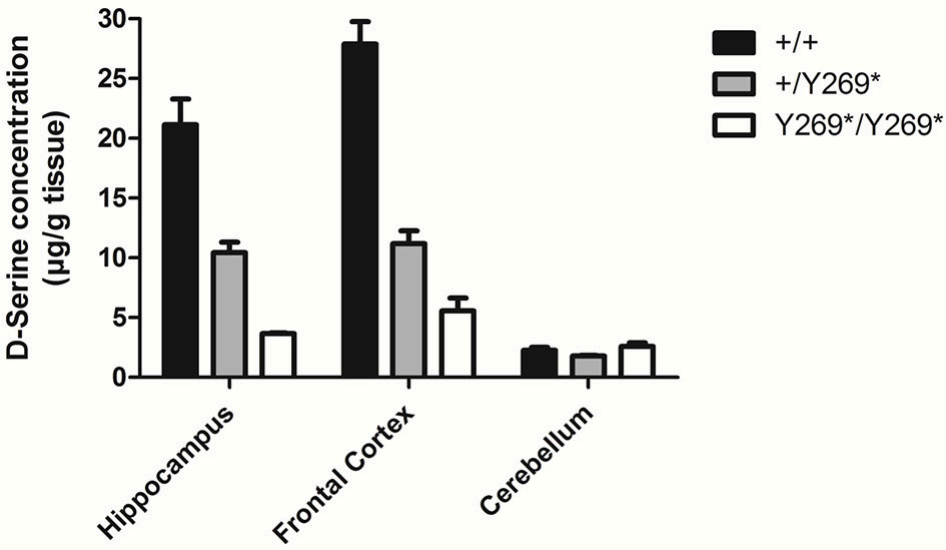
Levels of D-serine in frontal cortex, hippocampus and cerebellum in mice with a nonsense mutation of exon 9 of the gene for SR: wild type, (+/+), heterozygous (+/Y269*), and mutant (Y269*/Y269*) mice. Behavioral deficits (impairment in prepulse inhibition, sociability, and spatial discrimination) in the mutant mice were worsened by an NMDA receptor antagonist and ameliorated by D-serine or clozapine [adapted from Labrie et al. (6)].

Several amino acids have a chiral center and thus can exist as D- and L-isomers. For many years, it was thought that only the L-isomers of these amino acids existed in mammalian tissue. However, it was discovered in the 1990s that relatively large quantities of free D-serine exist in the mammalian brain (1–3), although at lower concentrations than L-serine (1–5). Free D-aspartate and D-alanine (Figure 1) are also present in brain at levels much lower than those of D-serine and of their respective L-isomers, but still measureable (2, 3, 5, 7–13). Interestingly, it has been reported that all three of these D-amino acids may contribute to brain function (14–22) and may be useful as adjuncts in the therapy of schizophrenia (4, 7, 10, 17, 20–22).

The focus of this review is on D-serine and its possible involvement with both schizophrenia and depression. D-Serine is a potent coagonist at the N-methyl-D-aspartate (NMDA) glutamate receptor and appears to have a major modulatory role in NMDA receptor-mediated neurotransmission, neurotoxicity, synaptic plasticity, and cell migration (5, 8, 15, 18–23). Considerable research now indicates that it may be a potential therapeutic agent and/or biomarker in both schizophrenia and major depressive disorder, as will be discussed in this review paper.

## Methods

Searches were done in PubMed and Web of Science covering the period 1990–2018 and the key phrases “D-serine and neuropsychiatric disorders,” D-serine and schizophrenia,” “D-serine and depression,” “D-amino acids in neuropsychiatric disorders,” and “D-serine and ketamine” were used in the searches. Only papers in English were used in the preparation of this review. The references obtained were screened by the authors to determine which would be best to put in this paper.

## D-Serine as a Possible Biomarker and/or Therapeutic Agent in Schizophrenia

There is now a large body of evidence supporting hypofunction of NMDA glutamate receptors in schizophrenia (24–27). Because D-serine is such a potent coagonist at the NMDA receptor, there has been a great deal of interest in its role in the brain. D-Serine is present in glia (mainly astrocytes) and neurons. It has been proposed as both a glial transmitter (28, 29) and a neurotransmitter (30), and this has resulted in considerable controversy [see (29, 30) for an interesting discussion of the relevant importance of glia and neurons in the actions of D-serine]. Wolosker etal. (30) have proposed that astrocytes synthesize L-serine which then shuttles to neurons to be converted to D-serine.

The NMDA glutamate receptor requires not only glutamate but a coagonist in order to be activated. For many years, it was thought that glycine was the coagonist and the site at which it acts on the NMDA receptor is termed the glycine binding site. There is now considerable evidence, including a regional distribution more closely resembling that of NMDA receptors than is the case with glycine (4) and a stronger affinity than glycine for the glycine binding site on the NR1 subunit of the NMDA receptor (31, 32), indicating that D-serine may be more important in this regard. Because of the large body of evidence indicating hypofunctioning of the NMDA glutamate receptor in schizophrenia, D-serine has become of great interest to many researchers, and it has been proposed as the primary NMDA receptor coagonist in the forebrain and hippocampus (14). Glycine and D-serine appear to act at different NMDA receptor populations, D-serine at synaptic receptors and glycine at extrasynaptic receptors (33). It has been proposed that synaptic NMDA receptors are neuroprotective and that extrasynaptic receptors may promote cell death (34).

A number of preclinical studies in rodents have demonstrated that lowering brain levels of D-serine by reducing the activity of serine racemase (SR), the enzyme responsible for catalyzing formation of D-serine from L-serine (e.g., Figure 1), can produce symptoms reminiscent of clinical symptoms in schizophrenia: stereotypies, cognitive deficits, disruption of prepulse inhibition (measure of sensorimotor gating), persistent latent inhibition (measures inhibitory learning and cognitive flexibility), and deficits in social interaction (6, 19–22, 35). It has also been reported that SR knockout mice show a reduction of basal NMDA receptor activity and reduced arborization and spine density in dendrites (36). Chronic D-serine reverses expression of activity-related cytoskeleton-associated protein (Arc) and causes partial rescue of dendritic abnormalities in the same model (36).

Spatial and reversal memory deficits in Sprague-Dawley rats treated with the NMDA receptor antagonist phencyclidine (PCP) at different developmental stages can be reversed with D-serine administration (37). Hagiwara etal. (38) conducted an experiment with SR-inhibited mice where D-serine was given as a supplement in the preadolescent phase, and this had some benefit in preventing adult onset psychosis, suggesting the possibility of using D-serine supplementation in early intervention in humans. Fujita etal. (39) reported that juvenile and adolescent rodents that had been exposed prenatally to maternal immune activation showed reduced expression of hippocampal NMDA receptor subunits and onset of cognitive deficits as adults; supplementing their drinking water with D-serine from P28 to P56 reduced cognitive deficits.

For further information about SR and D-amino acid oxidase (DAAO), see the next section.

## Formation and Catabolism of D-Serine and Relevance of this Metabolism to Schizophrenia

**Figure 2:**
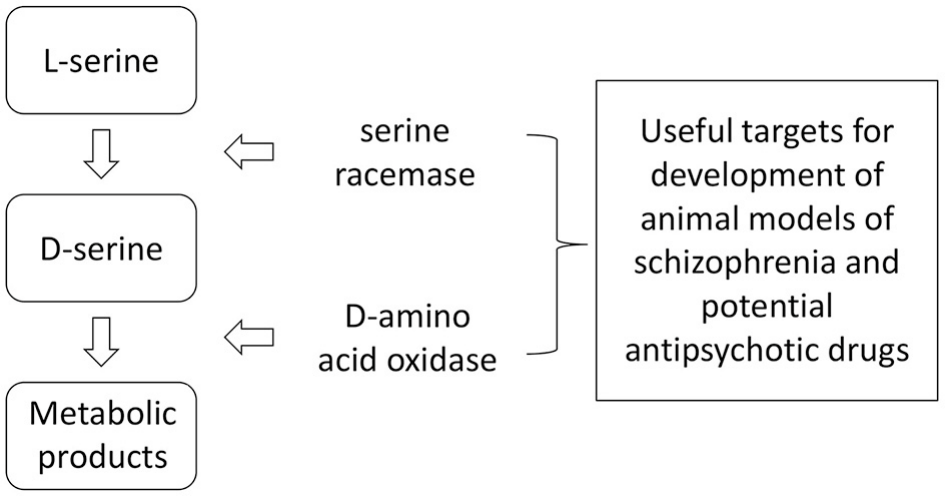
Anabolism and catabolism of D-serine. Because of the relatively large number of factors interacting with these enzymes, there are numerous potential targets for influencing brain levels of D-serine through genetic or pharmacological manipulations.

D-Serine formation from L-serine is catalyzed via the enzyme SR and its catabolism is catalyzed by DAAO (40–46) (Figure 2).

In the brain, SR and D-serine are found in the same regions, i.e., high levels in forebrain areas such as cortex and hippocampus and very much lower levels in the cerebellum and brain stem (35). Activity of SR can be modulated through various α-amino-3-hydroxy-5-methylisoxazole-4-propionic acid (AMPA) receptor-dependent, metabotropic mGluR2/3/5 receptor-dependent mechanisms, divalent cations and the adenosine triphosphate (ATP) pathway, suggesting potential targets by which D-serine concentrations could be enhanced (47–49). AMPA-induced postsynaptic membrane depolarization builds up intracellular calcium concentrations and stimulates SR with the soluble N-ethylmaleimide-sensitive factor attachment protein receptor (SNARE) (48). Ma etal. (50) reported SR interactions with stargazin and the scaffolding protein PSD-95 and suggested that these proteins regulate NMDA receptor-AMPA receptor cross-talk in neurons. Lin etal. (51) proposed an association of D-serine with PSD-95 and NMDA receptors in postsynaptic neurons and with stability of glutamatergic synapses during development of cortical synapses. Glutamate-receptor-interacting-protein (GRIP) forms a complex with mGluR2/3 receptors, and receptor activation changes the conformation of SR, altering its function and decreasing production of D-serine (49).

Single nucleotide polymorphisms in the gene encoding protein interacting with C-kinase 1 (PICK1), a component of protein kinase C signaling, have been reported to be associated with a higher risk of schizophrenia, and it has been suggested that this link could be mediated through the interaction with SR (52). Ma etal. (53) proposed that pathogenic disruption of Disrupted-In-Schizophrenia-1 (DISC1)-SR binding can produce schizophrenia-like behavior by depleting D-serine levels. DISC1, the perturbation of which has been implicated in the pathophysiology of a number of mental disorders, including schizophrenia, major depressive disorder and bipolar disorder (54), binds to SR and stabilizes it. Using a mouse model of schizophrenia, Ma etal. (53) found that mutant DISC1 results in SR degradation and a D-serine deficiency. Svane etal. (55) administered D-serine to male and female rats and measured expression of nitric oxide synthase1 adaptor protein (NOS1AP) (overexpressed in cortex of patients with schizophrenia), D2 receptors and DISC1 and found that it affects expression of these three genes in a sex-specific manner. Mustafa etal. (56) reported that NO S-nitrosylates SR, mediating feedback inhibition of formation of D-serine. Interaction of SR with Colga 3, a member of the Colgin subfamily A, may also be important since Colga 3 decreases the ubquitylation of SR, resulting in protection of the SR from degradation by the ubiquitin-proteasomal system (57). In their SR knockout mouse model (58), Balu and Coyle (59) observed reduced binding of cyclic adenosine monophosphate (cAMP)-responsive element binding (CREB) to the promoter regions of genes for three molecules implicated in the pathophysiology of schizophrenia, namely brain-derived neurotrophic factor (BDNF), microRNA-132, and Arc (60). In a recent review article, Wolosker has provided a useful table of regulators of SR activity and D-serine production (61).

In contrast to the distribution of SR and D-serine in the brain, DAAO is most abundant in the cerebellum and brain stem and at much lower concentrations in pre-frontal cortex, hippocampus, and substantia nigra (13, 35). It has been reported that the activity of DAAO in postmortem brain tissue from humans with schizophrenia is increased over that of controls (62, 63). Labrie etal. (41) found that genetic loss of DAAO reverses the schizophrenia-like phenotypes in mice displaying these behaviors because of a mutation in the NR1 subunit of the NMDA receptor. DAAO has been proposed to be regulated by the protein product of gene G72 (64–67), and variations in pLG72 have been associated with schizophrenia (68–72). There has been some discussion in the literature about the various functions of pLG72 in the brain and the molecular details of its interaction with DAAO, and these matters have been reviewed comprehensively by Pollegiani etal. (73). Hashimoto etal. (74) found that administering a DAAO inhibitor and D-serine simultaneously reduces prepulse inhibition seen in mice given dizocilpine, a non-competitive NMDA receptor antagonist. Other researchers have also co-administered DAAO inhibitors in conjunction with D-serine in preclinical studies and suggested that DAAO inhibitors could be useful in schizophrenia by reducing the required dose for D-serine (40, 74–77).

## Clinical Studies With D-Serine in Schizophrenia

Levels of D-serine have been reported to be decreased in cerebrospinal fluid (CSF) and blood of schizophrenia patients (18, 78–84). Hashimoto etal. (84) reported that serum levels of D-serine in schizophrenia patients are lower than in controls, while D-serine serum levels in mood-stabilized bipolar disorder patients are higher than those in controls, and they felt that this situation may reduce misdiagnosis between these disorders. Further, plasma levels of D-serine and the D-/L-serine ratio were increased significantly by clozapine in schizophrenia responders (85).

Clinical studies have been conducted in schizophrenia with D-serine alone and as an adjunct to antipsychotics. In a 4-week open-label study it was reported that D-serine at high dose (60 mg/kg/day) resulted in improved neurocognitive function (86). The same group did follow-up studies on D-serine (87, 88). In one investigation they did a double-blind, placebo-controlled, parallel group randomized clinical trial on negative symptoms in at-risk individuals, and D-serine was given at a dose of 60 mg/kg/day in divided daily doses for 16-weeks; it was concluded that D-serine should be useful for treatment of prodromal symptoms. In another double-blind study, they found that in schizophrenia patients treated with D-serine at 60 mg/kg/day, there was a significant improvement in mismatch negativity (MMN) (auditory mismatch), a neurophysiological biomarker for NMDA receptor activity. Ermilov etal. (89) compared high dose (3 g/day) D-serine with high dose olanzapine in treatment-resistant schizophrenia patients and concluded that a subgroup of patients could be maintained on D-serine. Interestingly, doses of D-serine as high as 4 g per day have been reported to cause no adverse effects (18).

Several clinical studies with D-serine have used it as a potential adjunctive drug (30–120 mg/kg/day) to antipsychotics. When added to non-clozapine antipsychotics in schizophrenia patients, D-serine has been reported to improve negative, positive, and cognitive symptoms (90–94) and to be relatively free of side effects even at high doses. A double-blind placebo-controlled study by Heresco-Levy etal. (91) examined the effect of supplemental D-serine in a group of patients stabilized on olanzapine or risperidone; at the end of 6-weeks of treatment, the D-serine intervention group demonstrated an improvement in cognitive, positive, and negative symptom domains and a significant alteration in depressive symptoms. Simultaneous measurement of serum amino acid concentrations did not detect any fluctuations of levels of any amino acids except D-serine. Meta-analyses of clinical trials comparing therapy with glycine modulatory site agonists, including D-serine, concluded that all of these NMDA receptor agonists significantly ameliorate symptoms in multiple domains, including cognitive and affective symptoms, when added to atypical antipsychotics except for clozapine (92–94). However, negative studies on D-serine adjunctive therapy in schizophrenia have also been reported in the literature (95–98). There have been inconsistent results as to the therapeutic benefit of D-serine used at 30 mg/kg/d to improve the negative and cognitive symptoms of the illness, with more consistent improvements found at doses of 60 mg/kg/d or higher. The higher doses of D-serine may be needed to adequately potentiate NMDA receptor-mediated activation of the receptor and to also achieve adequate serum levels and a subsequent predictive increase in brain concentrations.

It is interesting that D-serine addition to clozapine does not increase the efficacy of clozapine (99); this may be because clozapine releases D-serine and glutamate (100) and may have agonist or partial agonist activity at NMDA receptors (99–101). It is also possible that individuals on clozapine do not respond to D-serine since they are more often older and/or treatment-resistant (99).

One of the problems of treating symptoms of schizophrenia with D-serine is the fact that it is metabolized rapidly by DAAO, reducing its bioavailability and requiring administration of high doses, which could lead to peripheral neuropathies. There are also safety concerns that high concentrations of D-serine can cause potential nephrotoxicity related to its metabolism by DAAO as has been reported in rats that have developed acute tubular necrosis associated with higher doses of D-serine (102). However, in clinical studies that have administered D-serine (4-weeks duration) in doses up to 120 mg/kg/day, there have been no significant side effects (including nephrotoxicity) reported (86). In this particular study, one patient who received 120 mg/kg/d of D-serine did show 2+ proteinuria without glycosuria during the last week of treatment with no change in creatinine which completely resolved following the discontinuation of D-serine. In a 16-week intervention study by the same group using 60 mg/kg/d in clinically high-risk individuals, there were two patients who were discontinued in relation to abnormal renal values associated with treatment, with all other patients' renal abnormalities reported being resolved with continued treatment (87). The long-term side effects of D-serine beyond a 16-week treatment period are currently unknown. Additional studies that include longer time intervals should be conducted on this aspect of D-serine to ensure patient safety.

Studies on DAAO inhibitors indicate that they have limited effects on brain D-serine levels in mice, although there is some disagreement in the literature on this [see Guercio and Panizzutti (103) for a discussion]. However, as mentioned in the above section on enzymes involved in metabolism of D-serine, several researchers have now co-administered DAAO inhibitors in conjunction with D-serine in preclinical studies, and their findings suggest that DAAO inhibitors could be useful clinically in schizophrenia for reducing the required dose of D-serine and thus also reducing potential side effects associated with the administration of high D-serine (74–77). Sodium benzoate, a frequently used food preservative, is also a DAAO inhibitor and has been reported to have beneficial effects in schizophrenia when added to antipsychotics (104).

## D-Serine and Major Depressive Disorder

As indicated above, there is now a strong body of evidence indicating the possible involvement of D-serine in the etiology of schizophrenia and suggesting its potential as an antipsychotic or an adjunct to existing antipsychotics. It has thus been very interesting to see research results indicating that it may also have antidepressant effects and/or be a potential biomarker for depression and response to the antidepressant effects of ketamine. The possible mechanisms involved in the antidepressant actions of D-serine are the subject of another paper in this volume and of a paper by Chan etal. (105) and will not be discussed here in detail.

As mentioned above, in clinical studies investigating D-serine as an adjunct to antipsychotics, improvement in the affective symptoms of these patients was also often observed (e.g., 89–94). D-serine has now been reported to have antidepressant-like effects in rodent models of depression (105–107). Malkesman etal. (106) studied the behavioral effects in mice of a single, acute i.p. dose of D-serine in several tests of antidepressants, including the forced swim test, the female urine sniffing test following serotonin depletion and the learned helplessness paradigm and in mice lacking NR1 expression in excitatory neurons in the forebrain and found that D-serine gave a positive response in each of the behavioral tests but that the same behavioral tests conducted in mice lacking NR1 expression did not respond to D-serine. Otte etal. (107) studied transgenic mice in which SR was overexpressed using paradigms of anxiety, depression, and cognition. These studies were done in the absence and presence of D-serine in the drinking water. D-Serine administration resulted in a reversal of the findings in these tests, suggesting a reduced proneness toward depression-related behavior. Wei etal. (108) studied the effects of D-serine and ketamine on rats in the forced swim test and proposed that D-serine produces antidepressant-like effects through the same mechanisms as ketamine. Wang etal. (109) found that chronic social defeat stress (CSDS) in mice induces expression of the D-serine transporter through epigenetic activation and decreases levels of D-serine in the hippocampus, leading to depression-like behavior.

The DAAO inhibitor sodium benzoate has been reported to have beneficial effects in a depressed patient (110) and acute administration of D-serine to healthy subjects has been reported to reduce subjective feelings of depression and anxiety as measured by Visual Analog Scales (111). Ishiwata etal. (112) reported that CSF levels of D-serine in depressed patients correlate negatively with severity of depression. In a comprehensive study on 70 depressed patients, Hashimoto etal. (113) found increased serum levels of both serine enantiomers compared to controls, while serum levels of glycine, glutamate, and glutamine did not differ between the depressed patients and controls. In contrast, Mitani etal. (114) had reported that plasma levels of D- and L-serine in major depressive disorder (MDD) patients were the same as those in healthy controls and levels of glutamate, glutamine, and glycine were higher in MDD patients than in controls. Hashimoto etal. (113) suggested that differences in severity of the depression between the two studies might account for some differences. Because of their findings of increased serum levels of D- and L-serine in MDD patients and a higher ratio of L-serine to glycine levels in the MDD patients, Hashimoto etal. (113) suggested abnormalities in synthesis and catabolism of serine enantiomers in MDD.

It is now well-accepted that intravenous administration of the NMDA receptor antagonist ketamine can produce a rapid improvement of symptoms of depression (115, 116). As noted above, several studies indicate that D-serine, an NMDA receptor coagonist, can also produce antidepressant effects. Malkesman etal. (106) state that the two drugs may activate common, convergent downstream targets, resulting in similar effects on protein expression and producing comparable changes in synaptic plasticity and dendritic remodeling. There are some interesting interactions between D-serine and ketamine reported. There is now evidence indicating that D-serine may be a predictive biomarker for antidepressant response to ketamine, with low plasma D-serine levels predicting a response to (R,S)-ketamine (117). Singh etal. (118) examined the effect of (R)- and (S)-ketamine on Alanine, Serine, Cysteine Transporter 2 (ASCT2)-mediated transport of D-serine in adrenal pheochromocytoma PC-12 and human neural astrocytoma 1321N1 cells, and primary neuronal cells in culture and reported that (S)-ketamine decreased cellular export by selectively inhibiting ASCT2; the authors suggested that this interaction might represent a source of dissociative effects seen with (R,S)-ketamine. Singh etal. (118) also incubated PC-12 cells with a variety of ketamine metabolites and determined the IC_50_ values associated with attenuation of intracellular D-serine and proposed to use the findings to help in the design of more efficient modulators of D-serine.

## D-Serine and Cognition

Serious cognitive deficits can occur in a number of psychiatric disorders, including both schizophrenia and MDD. It has been reported that depletion of D-serine levels in brain diminishes long-term potentiation (LTP), which is associated with learning and memory (19, 20). Genetic inactivation of SR in a mouse model and an acute stress model reduced brain levels of D-serine and produced cognitive deficits (6, 119). Intraperitoneal injection of D-serine results in improved social memory in rats (120) and improved recognition and working memory in mice (121). In a rodent model study following development of offspring after prenatal maternal infection [using poly(I:C)], it was reported that supplementing offspring at juvenile and adolescent stages with D-serine reduced cognitive deficits in adulthood (39). Panizzutti etal. (122) studied the association between serum D-serine levels and the results of intensive cognitive training in schizophrenia patients and found that in those patients receiving this training increased D-serine levels were positively correlated with improved global cognition and verbal learning, but such associations were not apparent with glycine. Despite the interesting findings mentioned above, D-serine is not used clinically as a cognitive enhancer; in a recent review, Guercio and Panizzutti (103) have discussed various factors, including pharmacokinetics and possible side effects that must be studied in more detail in order to increase the efficacy of D-serine.

## Summary

In contrast to the usual situation with D-amino acids, D-serine is abundant in the brain and appears to have important neuromodulatory roles. D-Serine is more potent than glycine as a coagonist at the NMDA receptor, has a regional distribution in the brain that is similar to that of NMDA receptors and appears to be more closely associated with synaptic NMDA receptors than glycine (which is more closely associated with non-synaptic NMDA receptors). There has been considerable controversy about the concentration and function of D-serine in glia vs. neurons, but evidence in recent years indicates that neurons are more relevant to D-serine action than originally thought. Synthesis and catabolism of D-serine are catalyzed by SR and DAAO, respectively. Regulation of these enzymes by various factors is quite complex. Decreased levels of D-serine in schizophrenia have been reported in several studies. Increasing D-serine levels in the brain may be an effective adjunct to antipsychotic treatment. D-Serine administered alone or in combination with usual antipsychotics may be useful in treating schizophrenia, but the high doses required may cause peripheral neuropathies, although these have not been evident thus far using doses up to 4 g/day. Nonetheless, more studies at longer time intervals should be conducted on this aspect of D-serine to ensure patient safety. Several studies have been conducted with D-serine in combination with a DAAO inhibitor, with generally promising results obtained. Based on preclinical and clinical findings, D-serine also appears to have antidepressant properties. Several interesting pharmacodynamic and pharmacokinetic interactions between D-serine and ketamine have been reported, and, interestingly, evidence suggests that low plasma levels of D-serine may predict positive antidepressant response to ketamine. Several animal model and clinical studies also indicate that D-serine may be effective in reducing cognitive deficits, but that further study is necessary before considering it an effective cognitive enhancer for routine use in humans.

## Author Contributions

GB and M-AM wrote the first draft. All authors (GB, M-AM, MK, RT, NDM, SMD) contributed to the literature search and the editing of the manuscript.

### Conflict of Interest Statement

The authors declare that the research was conducted in the absence of any commercial or financial relationships that could be construed as a potential conflict of interest.
